# Micro Direct Methanol Fuel Cell Based on Reduced Graphene Oxide Composite Electrode

**DOI:** 10.3390/mi12010072

**Published:** 2021-01-11

**Authors:** Chaoran Liu, Sanshan Hu, Lu Yin, Wenli Yang, Juan Yu, Yumin Xu, Lili Li, Gaofeng Wang, Luwen Wang

**Affiliations:** 1 College of Electronic and Information Engineering, Hangzhou Dianzi University, Hangzhou 310018, China; liucr@hdu.edu.cn (C.L.); hss333999@163.com (S.H.); wenliyang2021@163.com (W.Y.); x923583923@163.com (Y.X.); lily5404@126.com (L.L.); gaofeng@hdu.edu.cn (G.W.); 2 Institute of Flexible Electronics Technology of THU, Jiaxing 314000, China; yinlu_ifet@163.com (L.Y.); yujuan@ifet-tsinghua.org (J.Y.)

**Keywords:** micro direct methanol fuel cell, composite electrode, catalyst carrier

## Abstract

The effect of an anode composite electrode on the performance of a micro direct methanol fuel cell (μDMFC) is analyzed from sample preparation configurations and discussed in detail, with a specific focus on the catalyst layer and the micro-porous layer on the anode composite electrode. This study investigates the effects of Pt content, Pt-Ru molar ratio, Nafion content, catalyst support, and preparation method in the catalyst layer, along with the carbon loading and polytetrafluoroethylene (PTFE) content in the micro-porous layer, on the performance of the anode composite electrode. The results show that the anode composite electrode delivers the best performance with 30% Pt content, a 1:1.5 Pt-Ru molar ratio, 10% Nafion content on reduced graphene oxide as the catalyst support. The synthesis is optimized with the impregnation reduction method using NaBH_4_ as the reducing agent, with the addition of 1.5 mg/cm^2^ carbon loading and 5% PTFE.

## 1. Introduction

A fuel cell is a type of power generation device that converts chemical energy into electric energy directly. It has the advantages of high energy conversion efficiency, high reliability, low noise, and is green and pollution-free [1,2]. At present, fuel cells have been used in the field of aerospace and submarines. Micro direct methanol fuel cells are widely used in micro robots, micro electronic equipment, micro medical devices, and personal mobile communication equipment. Therefore, they have an extensive future and wide application potential.

Although the performance of μDMFC is considerably more advantageous compared to other types of fuel cells, such as strong continuous power supply capability, high reliability, and convenient fuel replenishment, there are still many limitations preventing its wide adaptation for industrial and commercial use [3,4,5]. Firstly, for μDMFC, the noble metal platinum (Pt) is considered the best biofunctional catalyst for optimal cell performance. However, Pt is scarce, difficult to purify, and hence high-cost, which has a huge impact on the material cost of μDMFC [6]. The accurate control of Pt load in the cell to minimize cost while maximizing cell performance is one of the most urgent problems to be addressed. Secondly, during the reaction of μDMFC, methanol will permeate from anode to cathode through the proton exchange membrane. The bifunctional Pt catalyst is used on both the anode and cathode side of the membrane, and the methanol crossover will result in the undesirable anodic reaction taking place on the cathode, which forms negative potential and reduces the overall output power [7]. In addition, during the continuous consumption of methanol during the reaction, insufficient oxygen in the air electrode will result in the incomplete oxidation of methanol, generating carbon monoxide (CO) instead of carbon dioxide (CO_2_) and poisoning the catalyst by irreversibly attaching to the active sites of Pt, which greatly reduces the performance, stability, and life of the catalyst [8].

Of the utmost importance within the catalytic membrane, the anode electrode in the membrane directly contacts and reacts with methanol fuel; therefore, its performance has the greatest impact on the overall performance of the fuel cell. Previous studies have shown that different catalyst compositions and their supporting materials can significantly affect the performance of anode electrodes [9,10,11,12,13]. Among many materials, graphene, for its two-dimensional periodic structure, consisting of six-membered carbon rings, and its high conductivity properties, is considered one of the best options as catalyst supporting material for μDMFC.

In this work, the preparation process of the reduced graphene oxide catalyst prepared by the high-temperature polyol method was optimized, and the influence of the reduced graphene oxide catalyst prepared by different reduction methods on the performance of the methanol fuel cell was compared. In order to further improve the performance of the fuel cell, the composition of the reduced graphene oxide composite electrode was optimized, including the Pt-Ru molar ratio, Pt content, Nafion content, carbon loading, and PTFE content. This work provides a feasible strategy for improving the performance of μDMFC based on reduced graphene oxide composite electrodes.

## 2. Experiment 

### 2.1. Reagents and Materials

Conductive carbon paper (TGP-H-060, Toray Corporation, Tokyo, Japan), conductive carbon powder (Vulcan XC-72C, Cabot Inc, Alpharetta, GA, USA), chloroplatinic acid (H_2_PtCl_6_) (pure reagent for analysis), ruthenium(III) chloride (RuCl_3_) (pure reagent for analysis), sodium hydroxide (NaOH), methanol (pure reagent for analysis), 5 wt.% Nafion solution and 60 wt.% PTFE emulsion, Nafion 117 membrane (DuPont Inc., Seoul, Korea), graphene oxide (Laboratory Grade, Suzhou Hengqiu Branch Technology Co., Ltd, Suzhou, China), and isopropanol (pure reagent for analysis). IT8510 DC Electronic Load (Nanjing Edks Electronics Co., LTD. Nanjing, China), HS-XX40 Hot Press (Shanghai Hesen Electric Co., LTD. Shanghai, China), 040S Ultrasonic Cleaning Apparatus (Shenzhen Chaojie Technology Co., LTD. Shenzhen, China).

### 2.2. Preparation of Catalyst Layer Slurry

Firstly, 72 mg graphene oxide powder was added to 30 mL ethylene glycol, and the mixture was ultrasonically shaken for 1 h to make the graphene oxide uniformly dispersed to form 2.4 mg/mL graphene oxide dispersion. Subsequently, 6 mL 0.05 mM chloroplatinic acid solution and 9 mL 0.05 mM ruthenium chloride solution were added to the graphene oxide dispersion and sonicated for 1 h. After adjusting the pH to 10 with 0.5 M sodium hydroxide solution, the solution was processed according to two preparation methods: the first preparation method was to pour enough glycol as reducing agent into the reagent and stir at 160 °C for 6 h; the second method was to add sodium borohydride to the reagent as reducing agent and stir at room temperature for 6 h. After processing as above, the prepared solution was stirred and separated centrifugally. Next, the solution was dried in an oven at 80 °C for 12 h to obtain reduced graphene oxide-supported Pt/Ru catalyst powder. Finally, the catalyst powder was shaken with 0.5 mL Nafion solution and 5 mL isopropanol for 0.5 h to form a catalytic layer slurry.

### 2.3. Preparation of Electrode

The electrode of μDMFC mainly includes the diffusion layer and the catalyst layer. In this work, carbon papers with a size of 15 × 15 mm were prepared as the base of the diffusion layer. A micro-porous layer was fabricated on the carbon paper by the slurry spray method. The micro-porous layer slurry was prepared by dissolving 3.5 mg of carbon powder into 2 mL isopropanol and 0.5 mL PTFE emulsion. After ultrasonic agitation for 30 min, the micro-porous layer slurry could be prepared for use. The carbon loading and the PTFE content of the micro-porous layer were 1.5 mg/cm^2^ and 5%, respectively. The catalyst layer was fabricated on the micro-porous layer by the same spray method as the micro-porous layer. The preparation of the catalyst layer slurry was also similar to the micro-porous layer slurry. Catalyst powder (9 mg Pt/Ru for anode and 4.6 mg Pt for cathode) was added into 5 mL isopropanol and 0.5 mL Nafion solution by ultrasonic agitation over 30 min to prepare the catalyst layer slurry. The anode catalyst loading was 4.0 mg/cm^2^ and the cathode catalyst loading was 2.0 mg/cm^2^.

### 2.4. Preparation of Membrane Electrode Assembly (MEA)

**Figure 1:**
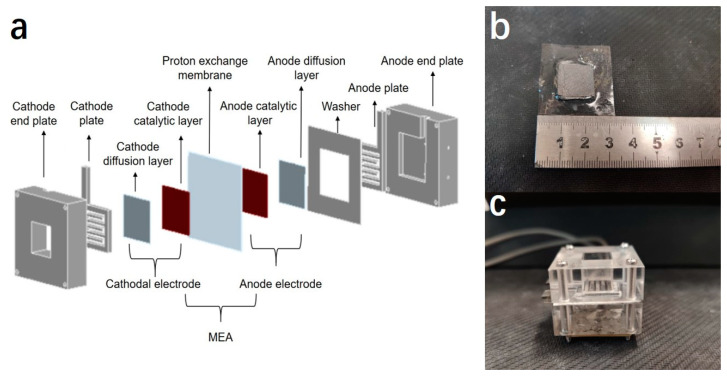
(**a**) Basic structure of the micro direct methanol fuel cell; (**b**) the prepared membrane electrode assembly (MEA); (**c**) assembled micro direct methanol fuel cell.

The membrane electrode assembly (MEA) was fabricated using the gas diffusion electrode (GDE) method. First, the Nafion117 membrane was used as the proton exchange membrane, which was pre-treated by boiling successively in 3 wt.% H_2_O_2_, deionized water, and 3 wt.% H_2_SO_4_ at 80 °C for 1 h each. Then, the pretreated Nafion 117 was sandwiched between the anode GDE and cathode GDE by hot-pressing at 135 °C and 0.6 MPa for 4 min to form the MEA, which is shown in Figure 1b.

### 2.5. Assembling, Activation and Performance Testing of Single Cells

The μDMFC in this work employed a traditional structure that consisted of two endplates, two current collectors, two gaskets and an MEA. Polymethyl methacrylate (PMMA) was used as endplate material due to its excellent mechanical characteristics. A square groove was fabricated on the anode endplate as the flow field area to place the anode current collector, while a square through-hole was fabricated on the cathode endplate to allow air transport into the cathode. All the endplates were fabricated by the machining process. Moreover, 316L stainless steel was used for current collectors because of its good electrical conductivity and corrosion resistance. A serpentine flow field was fabricated on the current collector by using wire cutting technology. Finally, the MEA, current collectors, gasket and endplates were assembled according to the sequence in Figure 1a. The whole structure of the μDMFC was clamped and fixed by screws, as shown in Figure 1c. Before the test, deionized water was fed into the anode for 12 h in order to infiltrate and activate the MEA. Next, 2 M methanol solution with a flow rate of 2 mL/min was injected into the anode flow field while operating at room temperature. The cell performance was recorded by DC electronic loading. The loading current started at 0 mA, and the voltage value was recorded every 5 mA to obtain the I-V and I-P curve.

## 3. Results and Discussion

### 3.1. Structural Characterizations of Graphene Oxide (GO) and Reduced Graphene Oxide (rGO)

**Figure 2:**
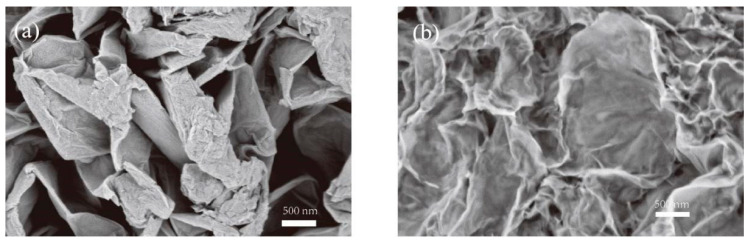
SEM images of (**a**) graphene oxide (GO), (**b**) reduced graphene oxide (rGO) nanostructures.

The graphene oxide before reduction treatment and reduced graphene oxide were characterized by SEM. As shown in Figure 2a, the SEM image of GO shows randomly aggregated crumpled layer structures due to deformation in the graphite structure upon exfoliation processes. The SEM image of rGO exhibits randomly crumpled wave-like structures, which reveals the presence of imperfections, breakage of crystals and a non-uniform layer because of the absence of oxygen-containing functional groups, reduced to a greater extent. 

### 3.2. Influence of High-Temperature Polyol Preparation Process on the Performance of Micro Direct Methanol Fuel cEll (μDMFC)

During the preparation process, catalytic metal elements, catalyst particle size and catalytic surface roughness would result in differences in catalysis performance. Ethylene glycol is generally used as a reducing agent in the high-temperature polyol method, as ethylene glycol can be easily oxidized to form strong reductive glyoxal and ethylene glycol (oxalic acid). However, various parameters in the preparation process, namely (1) pH, (2) heating temperature, and (3) the time of heating, have a great impact on the performance of ethylene glycol reduction. Thus, various parameters were closely studied in this work.

#### 3.2.1. The Effect of Reducing pH on the Performance of μDMFC

**Figure 3:**
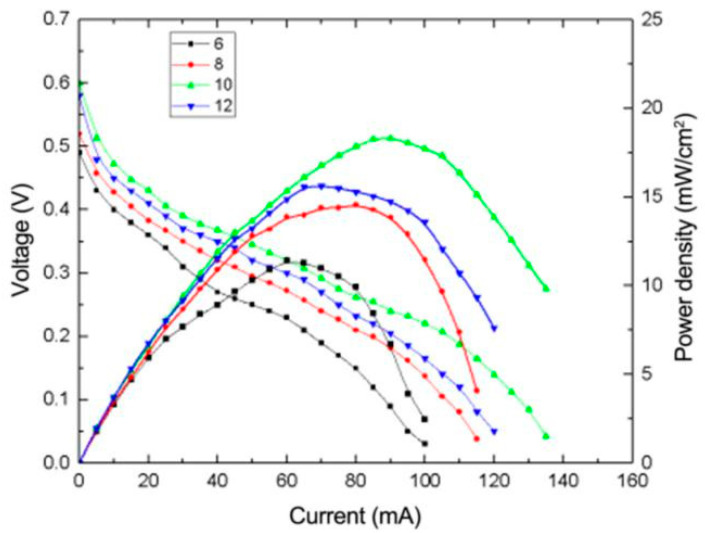
Effect of different pH values during the reduction process on micro direct methanol fuel cell (μDMFC) performance.

Figure 3 shows the polarization curve and power density curve of MEA prepared at different pH values in 2 M methanol solution. The pH of the solution is strongly acidic, which is around 2–3, before the addition of NaOH. In an acidic environment, H^+^ is more likely to react directly with glyoxal or oxalic acid, which result from the oxidation of ethylene glycol. Both glyoxal and oxalic acid have strong reducing properties. However, chloride and Ru^3+^ ions are less dissociated under a strongly acidic environment. Therefore, it is necessary to adjust the pH value of the reaction environment before reduction. In an alkaline environment, the OH^−^ can reduce and dissociate the chloride anion and Ru cation and allow the Ru^3+^ to be reduced by glyoxal or oxalic acid to form metal particles. It can be seen that when the pH is different, the performance of the fuel cell varies greatly. The environment is still acidic at pH 6, so the open-circuit voltage (OCV) and power of the fuel cell are considerably low (0.5 V), reaching the peak power density of 12 mW/cm^2^ at 60 mA. At pH 8, when the environment changed from weak acid to weak alkaline, the performance of the whole fuel cell improved significantly, reaching the peak power density of 14 mW/cm^2^. When pH = 10, the OCV and maximum power density of the fuel cell were the highest, reaching 0.6 V and 18 mW/cm^2^, respectively. From the data, we can deduce that a higher concentration of OH^−^ in the solution is suitable for the reduction reaction process, which allows for the full reduction of Pt and Ru; the best electrocatalytic performance for the electrooxidation of methanol can be obtained at pH value of 10. When the pH rises to 12, the performance of the fuel cell begins to decline. This may be due to the high concentration of OH^−^, causing the reducing agent to consume too much before reducing the Ru^3+^, reducing the reduction efficiency and resulting in a reduction in the utilization rate of Pt and Ru. Therefore, the optimal pH value for the impregnation reduction process is determined to be 10.

#### 3.2.2. Effect of Heating Temperature on the Performance of μDMFC

**Figure 4:**
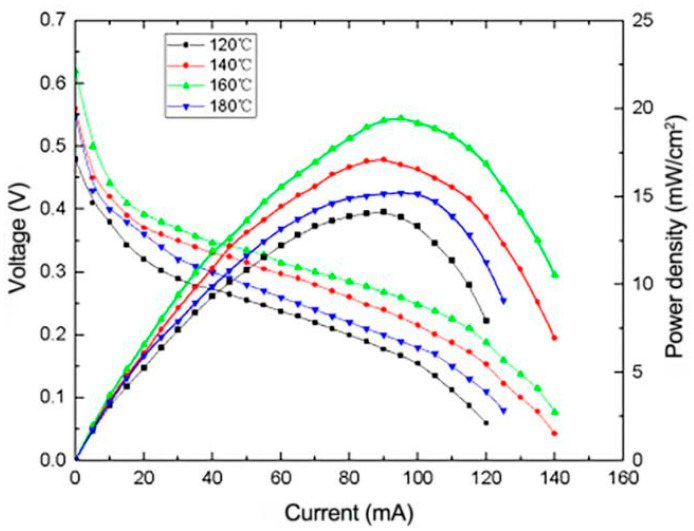
Effect of different heating temperatures on the performance of μDMFC.

Figure 4 shows the polarization curve and power density curve of MEA prepared at different reaction temperatures. The reduction of ethylene glycol can only occur at high temperature by the method of high-temperature polyol, so the temperature is generally set at 100–200 °C. Ethylene glycol is more likely to produce glyoxal or oxalic acid at high temperature. As shown in Figure 4, the optimum heating temperature is 160 °C, where the peak power density reaches 19.6 mW/cm^2^. As the heating temperature increases from 120 to 160 °C, the performance of the fuel cell increases gradually due to the accelerated rate of the production of glycolaldehyde or oxalic acid by ethylene glycol. The increased reduction reaction rate allows a higher amount of Pt and Ru to be loaded on the carrier. However, the performance of the fuel cell drops sharply when the temperature reaches 180 °C, which is deduced to be caused by the loss of ethylene glycol near its boiling point (197 °C) by evaporation [14]. As a result, the loss of the reactant resulted in the low yield rate of Pt and Ru reduction. Overall, the optimal reaction temperature is 160 °C in this experiment.

#### 3.2.3. The Effect of Heating Time on the Performance of μDMFC

**Figure 5:**
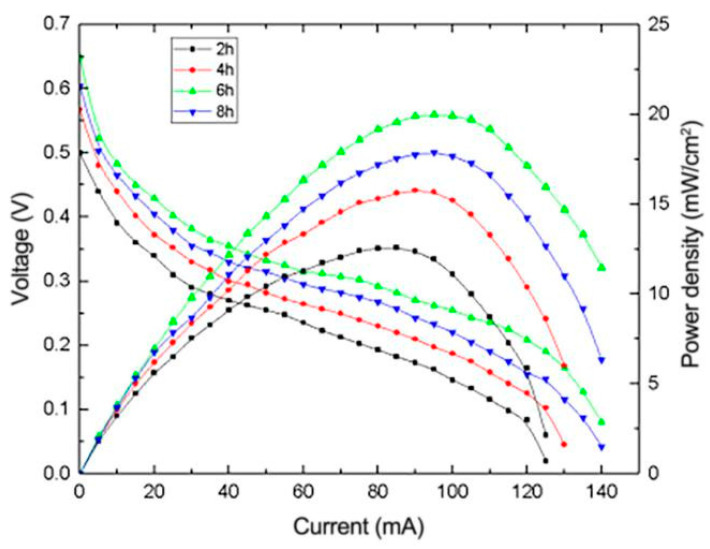
Effect of heating time on the performance of μDMFC.

Figure 5 depicts the I-V and I-P curves of μDMFC with different heating times in the catalyst preparation process. The cell with the heating time of 6 h exhibits the best performance when the pH and the heating temperature are set at 10 and 160 °C, respectively. It can be found that the cell performance increases continually with the heating time. This is due to the fact that the reduction reaction rate in the solvent is still rising and does not reach the maximum value during the first six hours. However, when the heating time is operated to increase continually and exceeds 6 h, the performance of the fuel cell drops obviously, which indicates that the Pt and Ru have reached the maximum value of reduction, and continuous high temperature makes the formed reduced graphene oxide loaded with Pt-Ru catalyst unstable, leading to degradation of cell performance [15].

**Figure 6:**
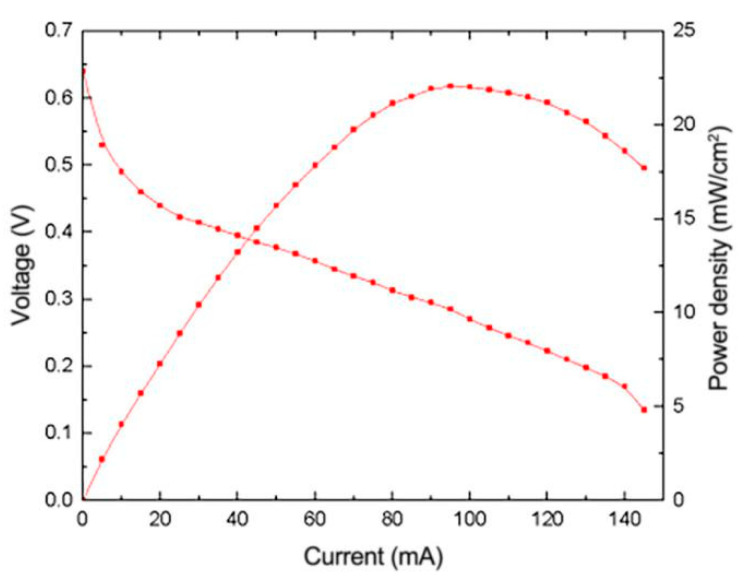
Performance curve of μDMFC prepared under optimal process parameters.

According to the above experiments, the pH value, heating temperature and heating time during the preparation and reduction process have a great influence on the performance of the composite electrode. According to the experimental results, the optimal pH value, the optimal heating temperature and the optimal heating time are 10, 160 °C and 6 h, respectively. As shown in Figure 6, under the optimal process parameters, the power density of μDMFC with the reduced graphene oxide composite electrode can reach 21.9 mW/cm^2^ under the high current load of 95 mA.

### 3.3. Effect of Impregnation Reduction Method on the Performance of μDMFC

**Figure 7:**
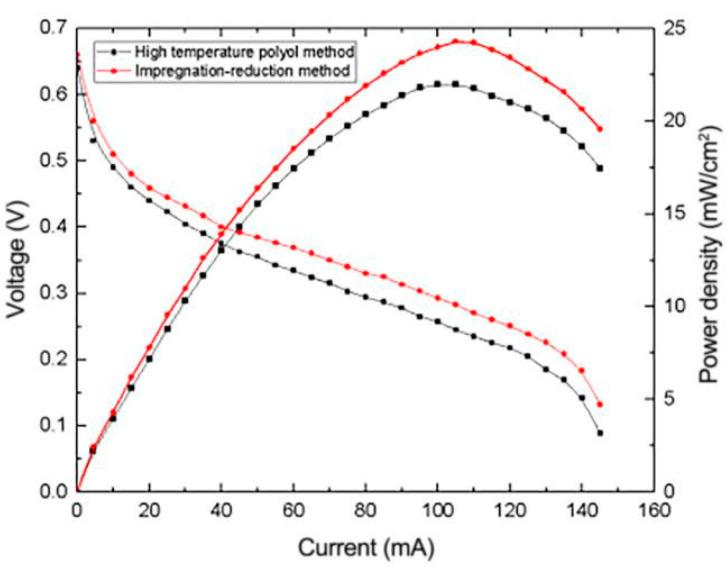
Effects of different preparation methods on the performance of μDMFC.

In this work, the effects of different preparation methods on the properties of the μDMFC were investigated; the catalyst was prepared by the high-temperature polyol method with ethylene glycol as the reducing agent and the impregnation reduction method with sodium borohydride as the reducing agent. An anode electrode was prepared and employed by μDMFC for testing. The results are shown in Figure 7.

It can be seen from Figure 7 that when the other preparation conditions and operating conditions are consistent, the cell performance with the impregnation reduction method is higher than when the high-temperature polyol method is used. The reduced graphene oxide composite electrode prepared by the impregnation reduction method reached the peak power density of 24.3 mW/cm^2^ at 105 mA loading current, which was 13.7% higher than that achieved by the high-temperature polyol method. This can indicate that the same amount of reducing agent NaBH_4_ has higher reducibility than ethylene glycol.

### 3.4. Effect of Composition of Catalytic Layer on the Performance of μDMFC

#### 3.4.1. Effects of Different Catalyst Carriers on the Performance of μDMFC

**Figure 8:**
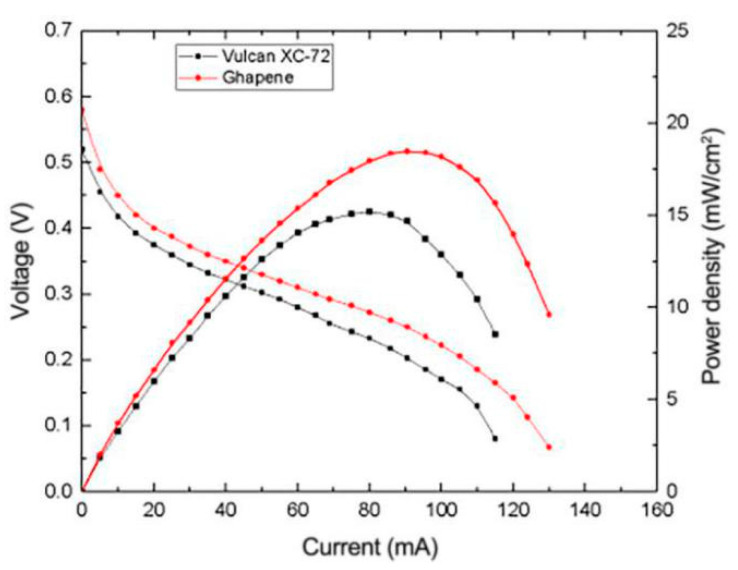
Effect of composition of catalytic layer on the performance of μDMFC.

In order to explore the effects of different catalyst carriers on the performance of µDMFC, reduced graphene oxide and the common Vulcan XC-72 carbon were selected as the carriers for comparison, and the results obtained are shown in Figure 8. It can be seen from the figure that µDMFC with composite electrodes using reduced graphene oxide as the carbon carrier have the better performance, with nearly 30% higher peak power density than that using Vulcan XC-72 carbon. It can be found that under the same amount of Pt load condition, reduced graphene oxide displays a better Pt loading characteristic due to its larger specific surface area compared to the traditional carbon carrier.

#### 3.4.2. Effect of Pt-Ru Molar Ratio on the Performance of μDMFC

**Figure 9:**
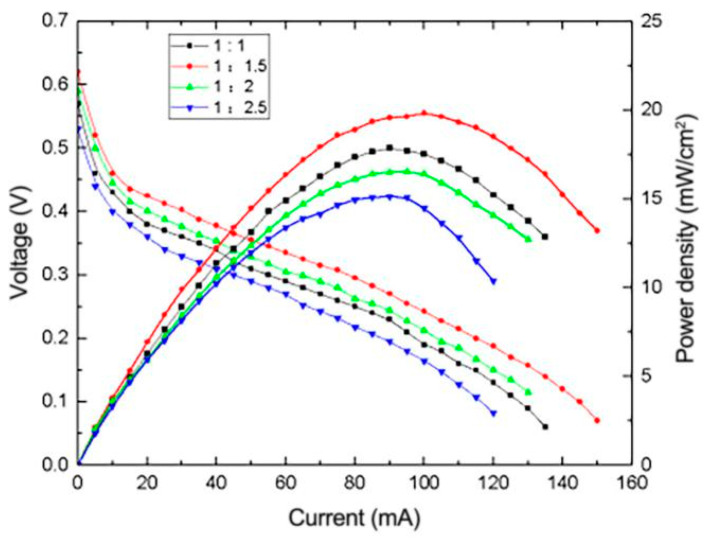
Effect of Pt-Ru molar ratio on the performance of μDMFC.

In the Pt-Ru catalyst system, Ru can more easily reduce OH^-^ in water than Pt, and it promotes the oxidation reaction of Pt intermediate products, which can effectively reduce CO adhesion on the surface of Pt due to incomplete oxidation, thereby enhancing the electrocatalytic performance of Pt [16]. However, excessive Ru will surround the surface of Pt particles, which will reduce the catalytic oxidation surface area of Pt particles. The influence of reduced graphene oxide composite electrodes with Pt-Ru molar ratios of 1:0.5, 1:1, 1:1.5 and 1:2 on the performance of μDMFC was investigated, and the results are shown in Figure 9. As shown in Figure 9, the μDMFC exhibits the best performance when the molar ratio of Pt:Ru is 1:1.5. When the molar ratio of Pt:Ru is 1:1, the content of Ru relative to Pt is insufficient and it is difficult to completely reduce the CO adhered on the surface of Pt. When the molar ratios of Pt:Ru are 1:2 and 1:2.5, the content of Ru relative to Pt is excessive, which reduces the catalytic oxidation surface area of Pt particles.

#### 3.4.3. Effect of Pt Content on the Performance of μDMFC

**Figure 10:**
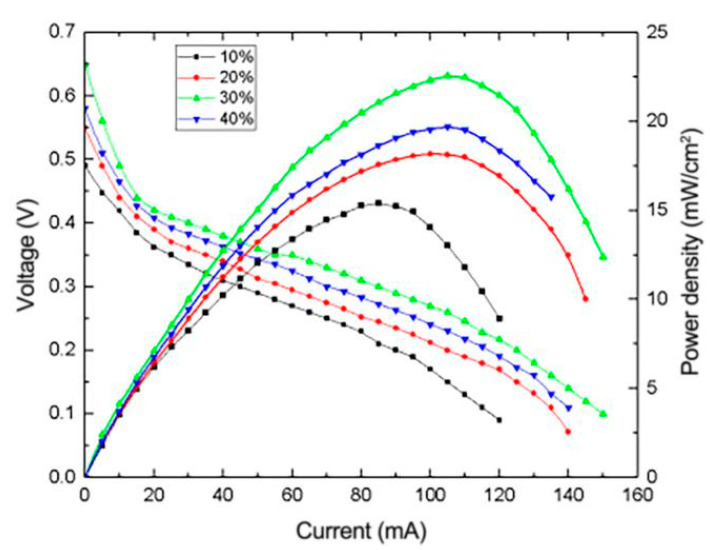
Effect of Pt content on the performance of μDMFC.

Pt is one of the main strong catalysts for the electrooxidation of methanol. Theoretically, the performance of the composite electrode will increase with the Pt content in the catalyst. However, excessive Pt content will cause cell performance degradation due to the agglomeration of Pt particles [17]. Therefore, the Pt content in the catalyst cannot be too high. In this paper, reduced graphene oxide composite electrodes with Pt content of 10%, 20%, 30% and 40% were prepared and employed into the μDMFC to investigate the effect of Pt content on the cell performance. The results obtained are shown in Figure 10.

As shown in Figure 10, when the Pt content is 30%, the prepared composite electrode exhibits the best performance. When the Pt content increases from 10% to 30%, the peak power density increases continuously, which indicates that Pt content has a positive impact on the performance of the fuel cell. When the Pt content is less than or equal to 30%, the dispersion of Pt in the carrier is not saturated, so there will be no agglomeration of Pt particles. However, when the Pt content increases to 40%, the dispersion of Pt in reduced graphene oxide will be saturated and excess Pt particles will easily agglomerate, resulting in low contact surface and lower cell performance.

#### 3.4.4. Effect of Nafion Content on the Performance of μDMFC

As a commonly used proton exchange membrane, Nafion has good proton conductivity and chemical stability. The Nafion membrane demonstrates obvious methanol permeation. Adding an appropriate amount of Nafion to the electrode catalyst can increase the active sites of Pt, improving the catalytic activity of the composite electrode for methanol oxidation [18,19]. Nafion also has a hole-making ability, which facilitates the formation of microporous channels in the catalytic layer to make the methanol solution uniformly dispersed in the catalytic layer and reduce the influence of mass transfer polarization. However, it is easy to form a Nafion-coated layer on the catalyst surface when the Nafion content is too high, reducing its electronic conductivity and effective reaction contact area. Moreover, the over-dense structure of the catalyst layer will prevent the methanol solution from infiltrating the whole catalyst layer, leading to a decrease in overall reactivity [20,21].

**Figure 11:**
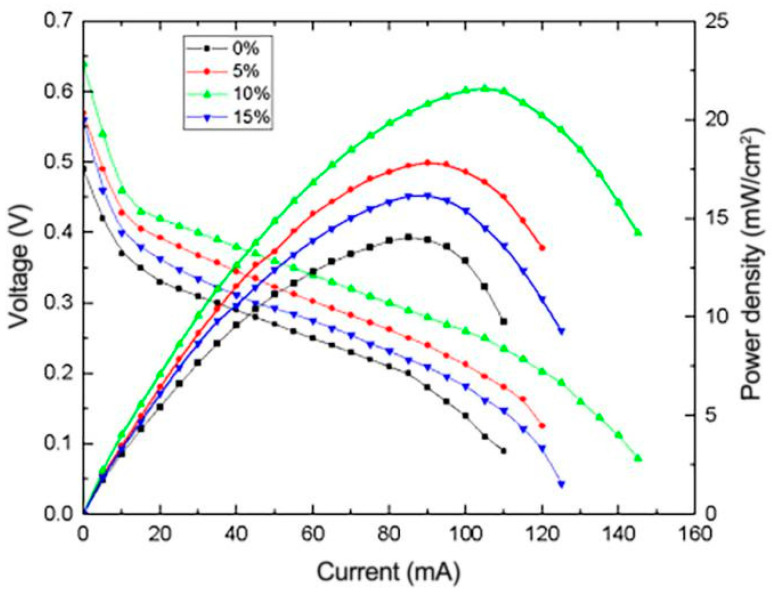
Effect of different Nafion content levels on the performance of μDMFC.

To investigate the effect of Nafion content on the performance of μDMFC, reduced graphene oxide composite electrodes with Nafion content of 0%, 5%, 10% and 15% were prepared and employed in the μDMFC for testing. Figure 11 illustrates the I-V and I-P curves of μDMFC with different Nafion content levels in the catalyst layer. It can be seen that the cell performance with Nafion content of 0%, 5% and 10% presents a gradient trend, which shows that the increasing of Nafion content in the range of 0% to 15% can effectively enhance cell performance. However, when the content of Nafion is over 10%, the performance begins to decline, indicating that Nafion gradually begins to form a coated layer on the surface of the metal catalyst particles. As the Nafion content continues to increase, the coated layer becomes thicker, which greatly increases the internal resistance and effective reaction area of the catalyst layer.

### 3.5. Effect of Micro-Porous Layer Composition on Performance of μDMFC

#### 3.5.1. Effect of Different Carbon Loading Amounts on the Performance of μDMFC

**Figure 12:**
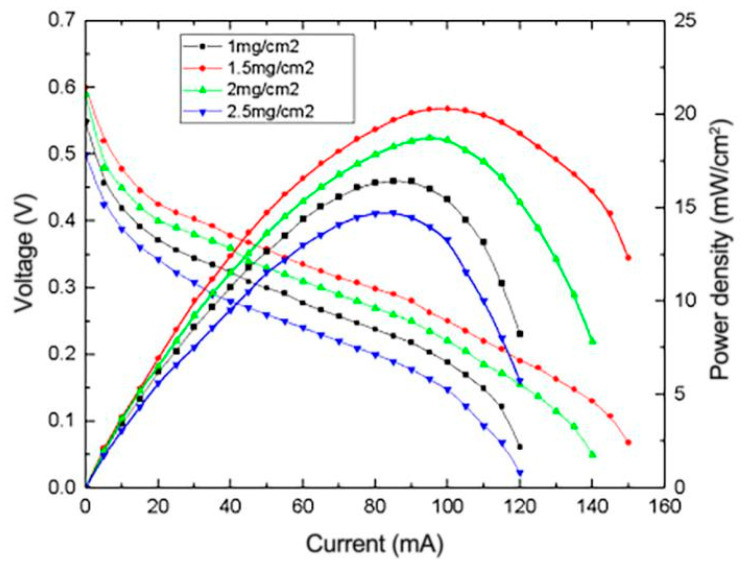
Effect of different carbon loading amounts on the performance of μDMFC.

Carbon loading refers to the amount of carbon in the diffusion layer of the composite electrode. It has a significant influence on the internal structure compactness of the prepared micro-porous layer and also has a great influence on the diffusion of methanol. An appropriate carbon loading amount can not only diffuse methanol evenly throughout the catalyst layer but also reduce the methanol crossover that is unfavorable to cell performance. In this experiment, composite electrodes with carbon loading amounts of 1, 1.5, 2 and 2.5 mg/cm^2^ were prepared, respectively, and employed by μDMFC for testing. The obtained I-P curves are shown in Figure 12.

From Figure 12, it can be seen that the optimum carbon loading of reduced graphene oxide composite electrodes is 1.5 mg/cm^2^. This result is different from that of traditional electrodes. In common commercial anode diffusion layers, optimal carbon loading is generally 2 mg/cm^2^, while the optimal carbon loading of the composite electrodes in this experiment is less than that of commercial electrodes. This shows that the reduced graphene oxide composite electrode prepared in this work has stronger electro-oxidation catalytic ability for methanol. When the carbon loading is 1 mg/cm^2^, peak power density is lower than that at 1.5 mg/cm^2^ carbon loading, suggesting that methanol penetration is severe at this value, causing an increase in the cathodic over-potential and reducing overall fuel cell performance. When the carbon loading is over 1.5 mg/cm^2^, fuel cell performance also declines, which is due to the excessive carbon loading seriously hindering the diffusion of the methanol solution. The diffusion rate is lower than the methanol consumption rate, resulting in a decrease in the methanol content on the catalytic layer, and the performance of the catalyst layer cannot be fully utilized. Therefore, the performance of the μDMFC decreases.

#### 3.5.2. Effect of Different PTFE Content Amounts on the Performance of μDMFC

**Figure 13:**
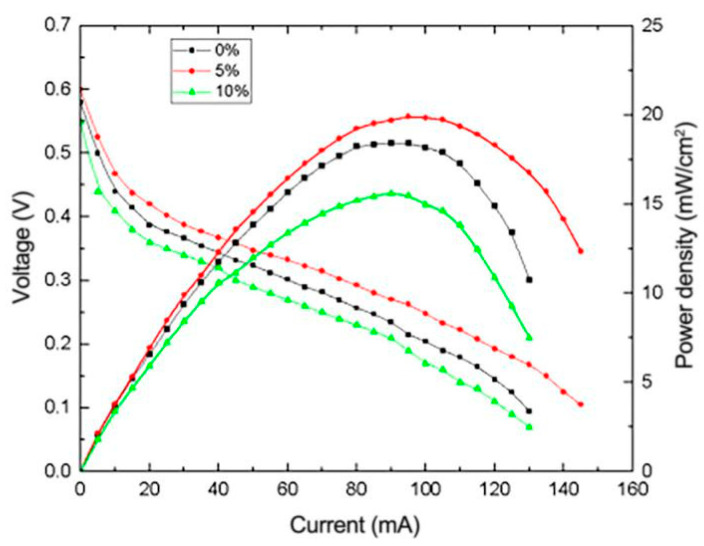
Effect of different PTFE content amounts on the performance of μDMFC.

PTFE is added to the microporous layer for two main purposes. One is that it has a certain viscosity, which can increase the tightness of the diffusion layer and the catalyst layer and reduce the ohmic polarization of the internal contact resistance; the other is that it can promote the formation of micropores in the microporous layer and facilitate the mass transfer of the methanol [22]. In order to explore the influence of PTFE content on fuel cell performance, reduced graphene oxide composite electrodes with PTFE content of 0%, 5% and 10% were prepared and employed by the μDMFC for testing. The obtained I-P curves are shown in Figure 13.

It can be seen that when the PTFE content reaches 5%, the fuel cell performance is better than that without adding PTFE. When the PTFE content reaches 10%, it can be clearly seen that the cell performance is lower than the performance without adding PTFE. This is because PTFE also has some hydrophobicity; the hydrophobicity of the electrode can hinder the diffusion of fuel and thereby lead to mass transfer polarization [23]. When the PTFE content is 5%, the positive effect of its cohesiveness and pore-making ability on the mass transfer polarization is greater than the adverse effect of hydrophobicity. Therefore, the μDMFC achieved the best performance with the PTFE content of 5%.

## 4. Conclusions

From the experimental data, it was concluded that the selection of materials in the synthesis of the catalyst and the composition of the anode composite electrode can significantly affect the performance of the fuel cell, all of which needs to be carefully controlled to ensure optimal performance. The parameters in the preparation process also have a great influence on the quality of the membrane, which also directly affects the performance of μDMFC. Under the experimental conditions, when the composition of the anode composite electrode includes a reduced graphene oxide catalyst carrier, 30% Pt content, 1:1.5 Pt-Ru molar ratio, 10% Nafion content, 5% PTFE content and 1.5 mg/cm^2^ carbon load, the micro direct methanol fuel cell demonstrates the best power performance. In this paper, the influence of the composition of the anode composite electrode on the micro direct methanol fuel cell is deeply studied, and a feasible strategy is provided for improving the performance of the methanol fuel cell based on the reduced graphene oxide composite electrode. For a complete membrane electrode, the cathode electrode is equally important, so further research can focus on the influence of the preparation method and composition of the cathode electrode on the performance of μDMFC.
